# A Snapback-Free and Low Turn-Off Loss 15 kV 4H–SiC IGBT with Multifunctional P-Floating Layer

**DOI:** 10.3390/mi13050734

**Published:** 2022-05-03

**Authors:** Xiaodong Zhang, Pei Shen, Zhijie Zou, Mingxin Song, Linlin Zhang

**Affiliations:** 1 School of Applied Science and Technology, Hainan University, Haikou 570228, China; zhangxiaodong@hainanu.edu.cn (X.Z.); zouzhijie1688@gmail.com (Z.Z.); 2 School of Mechanical and Electronic Engineering, Pingxiang University, Pingxiang 337055, China; peishen18@gmail.com; 3 Key Laboratory of Agro-Forestry Environmental Processes and Ecological Regulation of Hainan Province, School of Ecological and Environmental Sciences, Hainan University, Haikou 570228, China

**Keywords:** 4H–SiC, P-floating, snapback, turn-off loss

## Abstract

In this paper, a 4H–SiC IGBT with a multifunctional P-floating layer (MP-IGBT) is proposed and investigated by Silvaco TCAD simulations. Compared with the conventional 4H–SiC field stop IGBT (FS-IGBT), the MP-IGBT structure features a P-floating layer structure under the N-buffer layer. The P-floating layer increases the distributed path resistance below the buffer layer to eliminate the snapback phenomenon. In addition, the P-floating layer acts as an amplifying stage for the hole currents’ injection. The snapback-free structure features a half-cell pitch of 10 μm. For the same forward voltage drop, the turn-off loss of the MP-IGBT structure is reduced by 42%.

## 1. Introduction

The 4H–SiC insulated gate bipolar transistor (IGBT) has made significant progress in theoretical research for its low driving power and simple driving circuit. Silicon material semiconductor technologies have become more and more difficult for high voltage, high power, and high temperature applications [1]. For 10~20 kV class voltage, the 4H–SiC IGBT shows low conductive resistance and high current density compared with the 4H–SiC trench metal oxide semiconductor field-effect transistor (UMOSFET) due to the high carrier current densities which result from the holes injected from the p+ collector into the drift region during the forward conduction period. As a result, it has become a promising power semiconductor device. Many studies have attempted to produce a method of simulation/experiment design and fabricate a 4H–SiC IGBT device [2,3,4,5]; some researchers have focused on the ultra-low specific on-resistance [1,6,7,8], while others have aimed to solve the inherent tail current [9,10,11].

For high frequency, low turn-off loss, Si IGBT anode engineering is a commonly solution for excess carrier extraction, such as dual gates structures [12], shorted anode structures [13], striped anode structures [14], and any other structures summarized in [15]. For 4H–SiC IGBTs, the backside oxide etch has not been realized by experiment, and so the dual gates structure is not an available solution. The striped anode structure is an effect solution to reduce the turn-off loss. However, the shorted anode IGBT has not been reported.

Considering that the traditional shorted anode Si IGBT needs multiple MOS cells in parallel, the 4H–SiC IGBT with single cells and no snapback phenomenon during the turn-on period needs to be added to the research on device structure designs.

In this paper, a shorted anode type 4H–SiC IGBT realizing snapback-free phenomenon and low turn-off loss with multifunctional P-floating layer is proposed. During the period of device turnoff, the MP-IGBT uses the N+ collector region to quickly extract electrons from the N- drift region, which significantly reduces the device’s energy loss. In addition, the MP-IGBT can eliminate the snapback effect with only a 10 μm half-cell pitch and shows more uniform current distribution during the forward conduction period. It is worth mentioning that the P-floating layer increases hole currents at the bipolar mode and pinches off electron currents with the P collector region at the unipolar mode during the forward conduction period, which improves the device’s forward conduction capability. Because the simulation and manufacture of a conventional shorted anode 4H–SiC IGBT has not been reported, the proposed structure in this paper only compared to the conventional FS-IGBT.

## 2. Devices Structure and Mechanism

**Figure 1:**
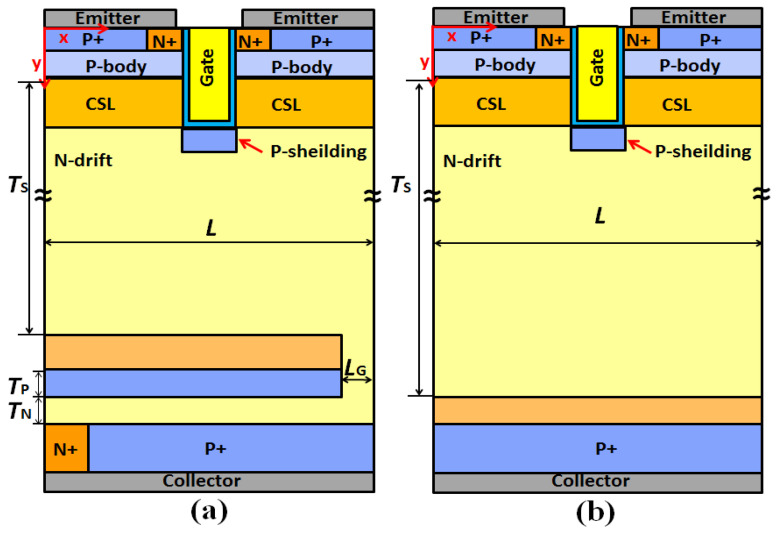
(**a**) Illustrative cross-sectional diagram of the MP-IGBT, and (**b**) of the conventional FS-IGBT.

Figure 1a shows a cross section of the MP-IGBT. This structure features a P-floating layer structure under the N-buffer layer and shorted anode structure. The P-floating layer is separated from the collector region by a part of the N-drift region. In addition, between each P-floating and N-buffer layer, there exists a gap in the conduction electron current during the turn-off period. Figure 1b shows the cross section of the FS-IGBT.

**Figure 2:**
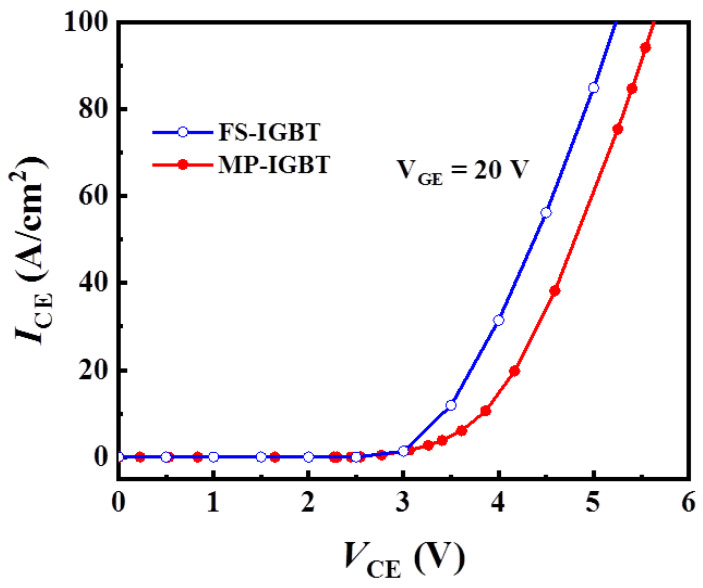
Comparison of the forward *I*–*V* characteristic curves of the MP-IGBT and the FS-IGBT.

Figure 2 shows the forward *I*–*V* curves of the MP-IGBT and the conventional FS-IGBT at the temperature of 300 K. It is obviously that the MP-IGBT shows no snapback phenomenon, but its forward conduction voltage drop at 100 A/cm^2^ is larger than that of the conventional FS-IGBT with a 10 μm half collector length. This is due to the electron current of the MP-IGBT partially flowing into the N+ collector region during the forward conduction mode, so the conductivity modulation effect is weaker than that of the conventional FS-IGBT [16,17].

**Figure 3:**
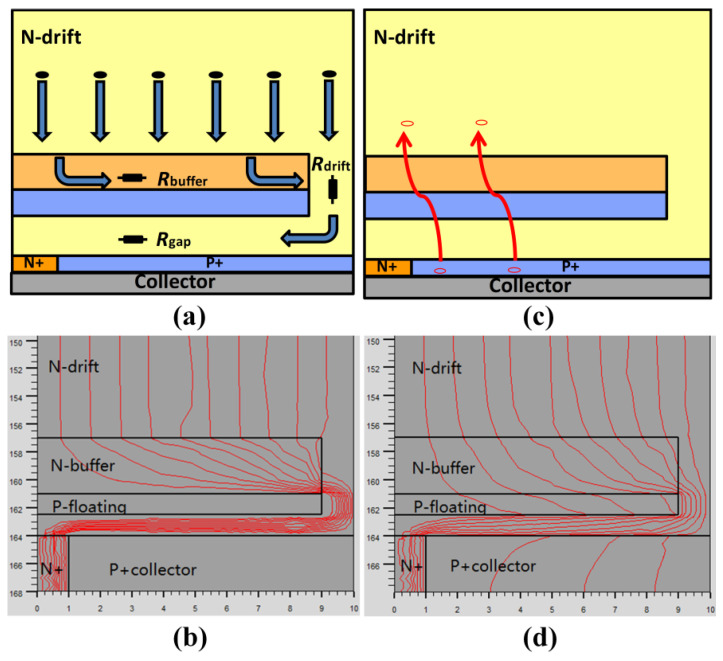
(**a**) Electron conduction path. (**b**) Hole conduction path. Current flowlines at the collector side of the MP-IGBT in (**c**) the unipolar mode and (**d**) the bipolar mode.

Figure 3 illustrates the electron and hole currents flow rules during the turn-on period. Figure 3a,b shows the electron currents flowing into the N-collector around the P-floating layer in the MP-IGBT in the unipolar mode, demonstrating that the P-floating layer enlarges the electrons’ flow path effectively.

For strip cells of Figure 1a, a simple model for the snapback voltage is based on [18]

(1) $$V_{\mathrm{SB}}=[1+\frac{R_{\mathrm{drift}}+R_{\mathrm{channel}}}{R_{\mathrm{buffer}}\cdot (L-L_{G})}]\cdot V_{\mathrm{critical}}$$

where *V*_SB_ is the snapback voltage at which the device switches from the unipolar mode to the bipolar mode. *R*_drift_ and *R*_channel_ are the drift region resistance and the channel resistance, respectively. *V*_critical_ is the critical voltage for the P+ collector/N-buffer junction initiating inject holes, *R*_buffer_ is the N-buffer resistance of the conventional shorted anode IGBT structure.

For the MP-IGBT, the P-floating layer prevents electrons from flowing toward the N-collector, enlarging resistance to suppress *V*_SB_ by extending the length of the trace (*L* − *L*_G_).

Figure 3c,d shows the hole currents flowing through the P-floating layer in the bipolar mode. Different from the unipolar mode, a part of current flow lines is from the P-collector during the bipolar mode.

## 3. Simulation and Discussion

Silvaco TCAD is used as a numerical simulation analysis tool to demonstrate the characteristics of the MP-IGBT. During simulation, the structure parameters of the two devices with an off-state blocking voltage in 15 kV are listed in Table 1. The parameters used in this paper are referred to in [19,20,21].

The gate oxide thickness (*T*_ox_) is 50 nm and the gate trench depth (*D*_G_) is 5 μm. During the simulation, the MOS cell pitch (*L*_M_) is set to 10 μm. For the trench gated 4H–SiC IGBT, *L*_M_ can shorten to 4 μm to promote MOS electron current density, or use injection enhancements with the P-floating region. The gap between the P-floating layer and the collector region (*T*_N_) is 1.5 μm, and the thickness (*T*_P_) and length of the P-floating layer (*L*_P_) are 1.5 and 9 μm, respectively, eliminating the snapback effect of the proposed structure. In order to improve the conductivity modulation effect, these P collector regions’ thicknesses are 4 μm.

**Figure 4:**
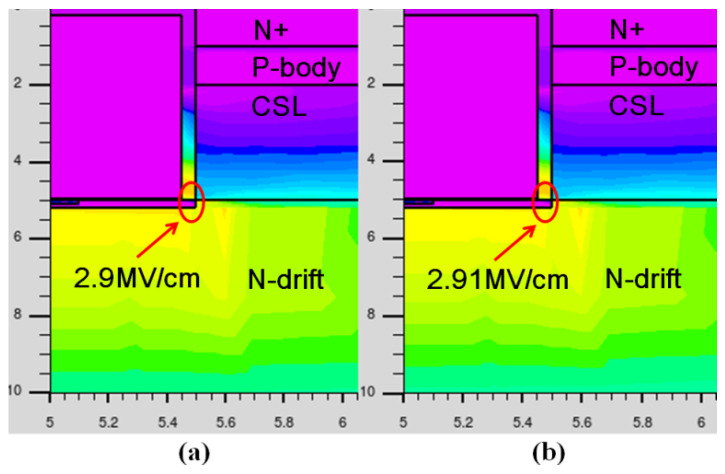
Electric field distribution in the top-side structure at V_CE_ = 15 kV. (**a**) FS-IGBT. (**b**) MP-IGBT.

Figure 4 shows the electric field distribution in the MP-IGBT at avalanche breakdown, where the collector-emitter voltage (*V*_CE_) is biased at 15 kV, and residual electrodes are connected to the ground. Due to the similar physical parameters of these two devices in the MOS structure and n-drift region, the blocking abilities of the MP-IGBT and the FS-IGBT are almost identical. 

**Figure 5:**
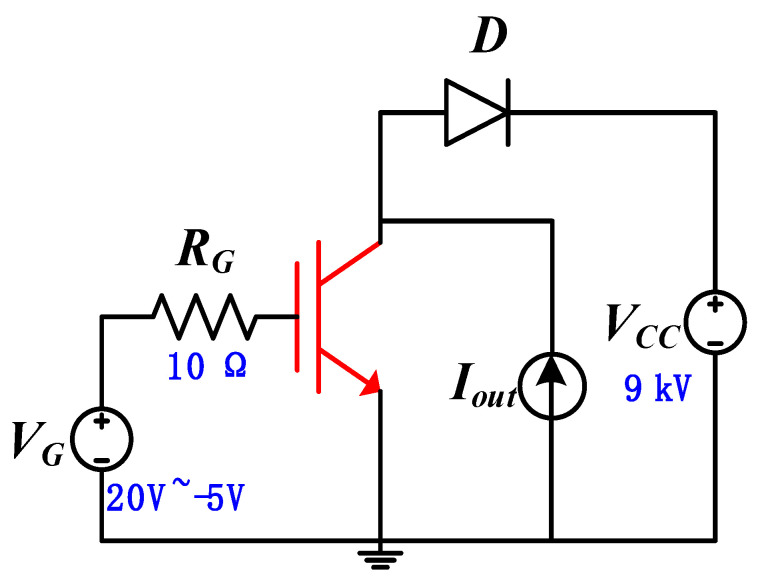
Inductive load circuit used in the switching simulations.

Similar to the conventional Si IGBT, the 4H–SiC IGBT also features a long tail current. Considering the high bus voltage, the 4H–SiC IGBT dissipated more energy than the Si IGBT. Therefore, it is an important issue to be solved. Figure 5 shows the inductive load circuit modeled by a constant current source (*I*_out_), a dc clamping voltage (*V*_CC_), and an ideal diode (*D*). The current source and clamping voltage are set to 1 × 10^−5^ A and 9 kV (60% of the breakdown voltage), respectively. For simplicity, the device’s active area is 1 × 10^−7^ cm^2^, making the current density flowing through the device 100 A/cm^2^. The gate resistor *R*_G_ is 10 Ω, and the gate voltage changes from 20 to −5 V. During this simulation, the diode is an ideal element.

**Figure 6:**
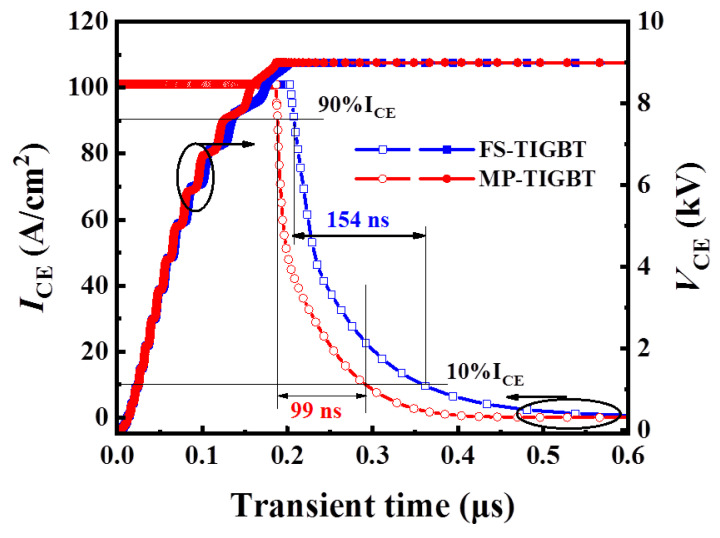
Turn-off voltage and current waveforms of the MP-IGBT and FS-IGBT, respectively.

Figure 6 shows the turn-off curves of the MP-IGBT and the conventional FS-IGBT of 100 A/cm^2^ at 300 K temperature. The half collector length used in the transient simulation of the two structures is 10 μm. When the forward conduction voltage is 6.175 V, the turn-off current transient time of the MP-IGBT and the conventional FS-IGBT are 99 ns and 154 ns, respectively. It can be seen that the MP-TIGBT shows shorter turn-off time than the conventional FS-IGBT.

**Figure 7:**
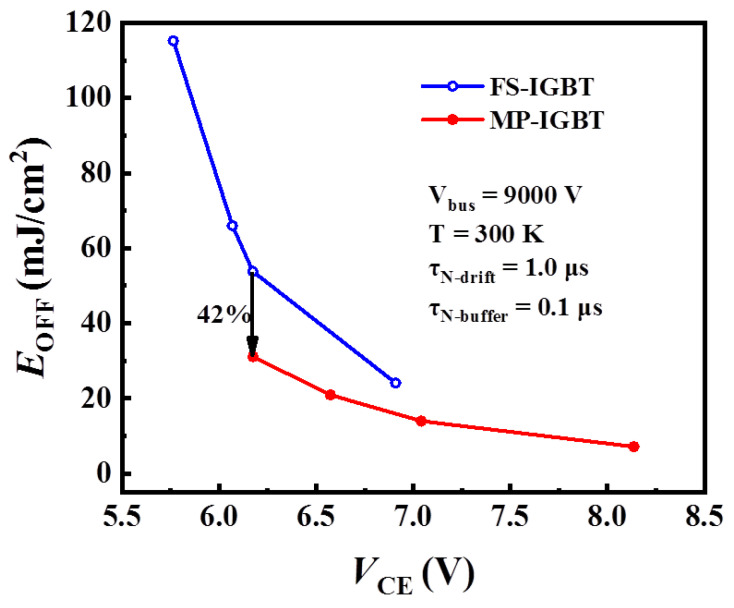
*E*_OFF_–*V*_CE_ relationships of the MP-IGBT and the FS-IGBT.

Figure 7 shows the tradeoff curves between *E*_OFF_ and *V*_CE_ for the MP-IGBT and the conventional FS-IGBT at 100 A/cm^2^ current density at 300 K temperature. In essence, the MP-IGBT and the conventional FS-IGBT use the same cathode structure. However, the MP-IGBT shows a better tradeoff relationship. At the position of *V*_CE_ = 6.17 V, the *E*_OFF_ of the MP-IGBT and the conventional FS-IGBT are 31.12 and 53.82 mJ/cm^2^, respectively. The MP-IGBT shows an *E*_OFF_ 42% lower than the conventional FS-IGBT structure. This is owing to the N+ collector region used in the bottom of the MP-IGBT. During device turnoff, electrons and holes are extracted away from the device under the high electric field, electrons flow toward to the bottom of the device, and holes run in the opposite direction to electrons. Compared with the P collector region, the N collector region can extract electrons more easily. As a result, the MP-IGBT shows lower energy loss.

**Figure 8:**
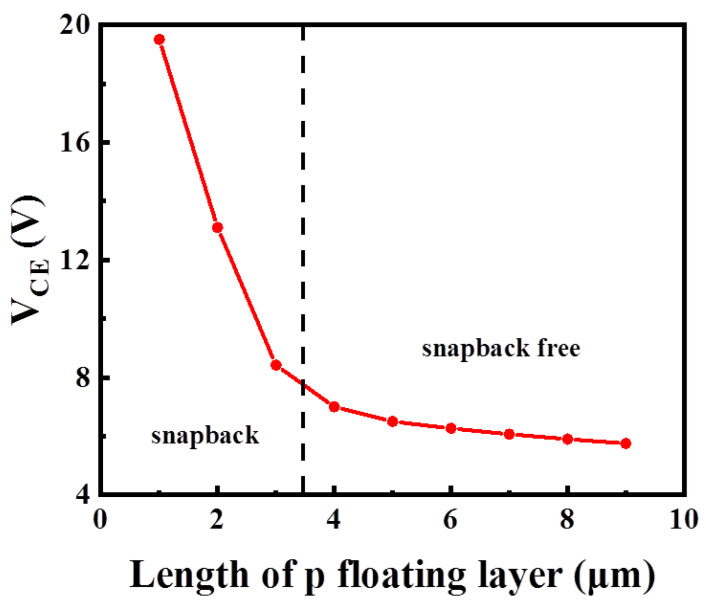
Dependence of *V*_CE_ on the length of P-floating layer. A longer P-floating layer contributes to lower *V*_CE_.

The P-floating layer under the N-buffer layer is used for suppressing the snapback effect of the MP-IGBT during the turn-on period. Figure 8 shows the relationship between the snapback effect and the length of the P-floating layer. As the length of the P-floating layer decreases, the *V*_CE_ increases. When the length of the P-floating layer is shorter than 4 μm, the snapback effect appears. Moreover, when the length of the P-floating layer is greater than 4, the snapback effect does not appear. This is due to the electron conduction path being pinched-off by the P-floating layer and the p collector region, so the rest of the electron path is enlarged.

**Figure 9:**
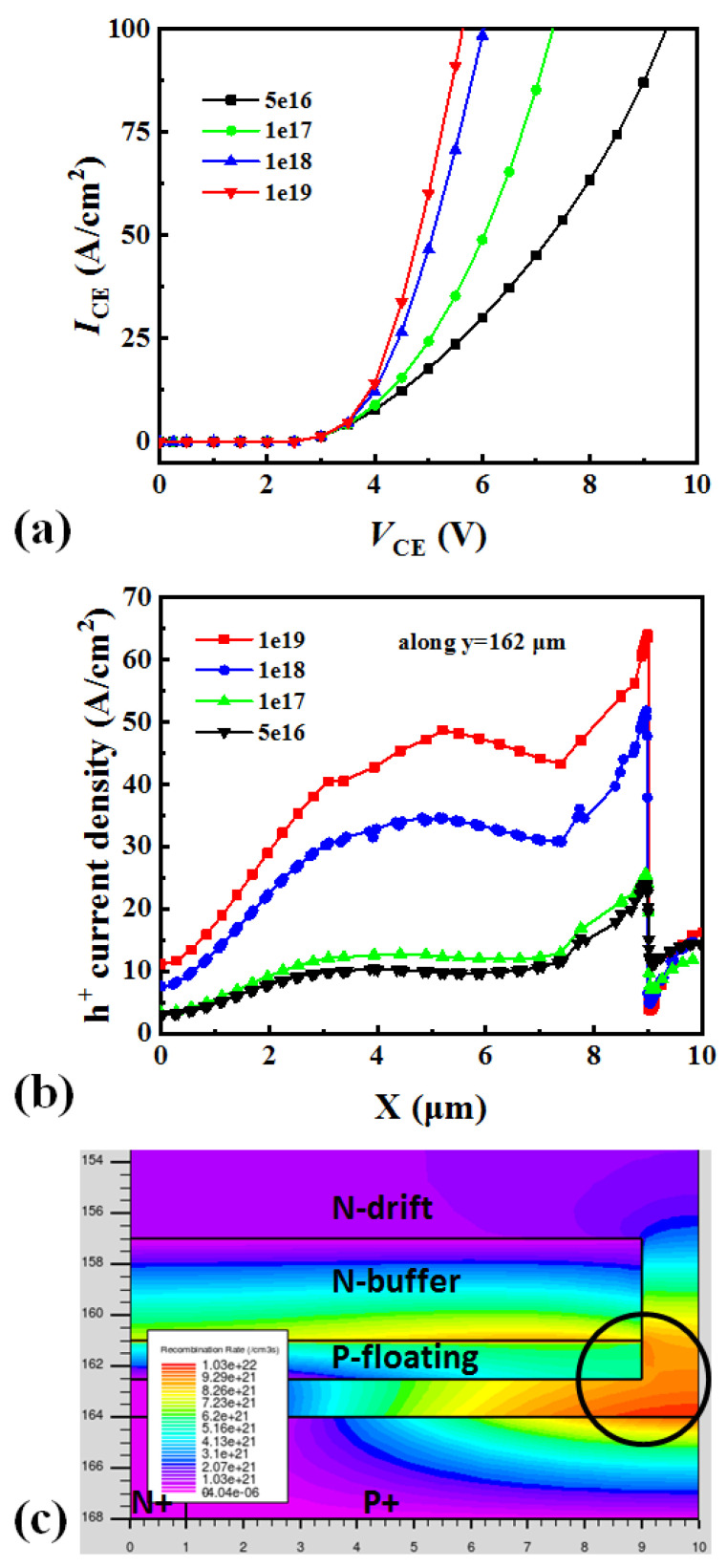
(**a**) Dependence of *I*-*V* curves on the doping concentration of P-floating layer. (**b**) Dependence of hole current density along y = 162 μm during on-state. (**c**) The schematic of recombination rate at the bottom-side structure.

Figure 9a shows the influence of the doping concentration of the P-floating layer of the MP-IGBT during the on-state period. Because the P-floating layer does not connect to the collector electrode, this layer does not inject holes into devices. As the doping concentration increases, the *V*_CE_ decreases. This is mainly due to the P-floating layer acting as the hole currents’ amplification stage. Figure 9b explain the phenomenon in Figure 9a by analysis the hole current density along the P-floating layer (in Figure 3b along y = 162 μm). Figure 9b shows the higher doping concentration of P-floating layer, the higher hole current density in device. The location of x = 9 (in Figure 3b) have the highest hole current density, this is due to the low doping concentration of N-drift region have low barrier to hole. However, the position of x = 9~10 μm shows low hole current density. This can be explained by Figure 9c. Figure 9c shows the recombination rate near the collector side. The electron and hole currents are recombined at the position in the circle marked in Figure 9c, so there are fewer hole currents injected into the devices.

**Figure 10:**
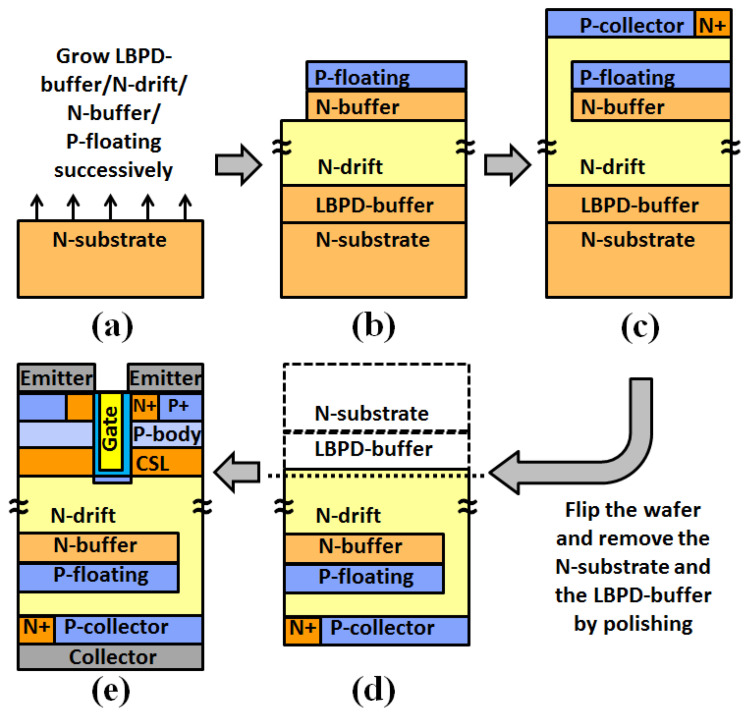
(**a**) The free-standing 4H–SiC N-substrate. (**b**) Forming LBPD-buffer, N-drift, N-buffer, P-floating. (**c**) The N-drift, P+, and N+ collector regions are formed by epitaxy and ion implantation. (**d**) Flip the wafer and remove the N-substrate and the LBPD-buffer by chemical mechanical polishing. (**e**) The finished MOS structure and electrodes.

The process flow of the MP-IGBT device is shown in Figure 10. The process for fabricating a high voltage n channel IGBTs on a free-standing 4H–SiC epilayer is used for building this device [11,22,23], and the process flow of the flip-type is also used [11,24,25]. Figure 10a shows the carbon face wafer of the N-substrate, and then the low-basal-plane-defect (LBPD) buffer, N-drift, N-buffer, P-floating are grown on the N-substrate that were illustrated in Figure 10b. As the N-drift, P+, and N+ collector regions are formed by epitaxy and ion implantation in Figure 10c, the wafer is then flipped and the N-substrate and the LBPD-buffer removed by chemical mechanical polishing to form Figure 10d. The MOS structure and electrodes of the fabrication processes are finished on the top surface of the N-drift layer as shown in Figure 10e.

## 4. Conclusions

This paper proposed a 15 kV 4H–SiC IGBT with the P-floating layer under the N-buffer layer. The P-floating layer acts as the hole currents’ amplification stage and suppresses the snapback effect during the turn-on period. The results of a comparative study have shown that the MP-IGBT can reduce the turn-off energy loss (*E*_OFF_) and suppress the snapback effect. The MP-IGBT features lower leakage current than the FS-IGBT, and the MP-IGBT shows an *E*_OFF_ 42% lower than the conventional FS-IGBT structure. 

## Figures and Tables

**Table 1:** Device parameters specification.

Parameters	MP-IGBT	FS-IGBT
MOS cell pitch (μm), L_M_	10	10
Gate oxide thickness (nm), T_ox_	50	50
Gate trench depth (μm), D_G_	5	5
Drift region doping (cm^−3^), N_d_	4.5 × 10^14^	4.5 × 10^14^
N-buffer doping (cm^−3^), N_Nb_	1 × 10^17^	1 × 10^17^
N-buffer thickness (μm), T_Nb_	4	4
P-collector doping (cm^−3^), N_Pb_	1 × 10^19^	1 × 10^19^
P-collector thickness (μm), T_Pb_	4	4
N-collector length (μm), L_N_	1	-
Half collector length (μm), L	10	10
N-drift thickness (μm), T_S_	155	155
CSL doping (cm^−3^), N_CSL_	1 × 10^15^	1 × 10^15^
P-base doping (cm^−3^), N_base_	4 × 10^17^	4 × 10^17^
P-floating layer thickness (μm), T_pf_	1.5	-
Length of P-floating layer (μm), L_pf_	1~9	-
